# Robust development of synfire chains from multiple plasticity mechanisms

**DOI:** 10.3389/fncom.2014.00066

**Published:** 2014-06-30

**Authors:** Pengsheng Zheng, Jochen Triesch

**Affiliations:** Frankfurt Institute for Advanced StudiesFrankfurt am Main, Germany

**Keywords:** synfire chain, recurrent neural network, network self-organization, spike-timing-dependent plasticity, homeostatic plasticity, network motif

## Abstract

Biological neural networks are shaped by a large number of plasticity mechanisms operating at different time scales. How these mechanisms work together to sculpt such networks into effective information processing circuits is still poorly understood. Here we study the spontaneous development of synfire chains in a self-organizing recurrent neural network (SORN) model that combines a number of different plasticity mechanisms including spike-timing-dependent plasticity, structural plasticity, as well as homeostatic forms of plasticity. We find that the network develops an abundance of feed-forward motifs giving rise to synfire chains. The chains develop into ring-like structures, which we refer to as “synfire rings.” These rings emerge spontaneously in the SORN network and allow for stable propagation of activity on a fast time scale. A single network can contain multiple non-overlapping rings suppressing each other. On a slower time scale activity switches from one synfire ring to another maintaining firing rate homeostasis. Overall, our results show how the interaction of multiple plasticity mechanisms might give rise to the robust formation of synfire chains in biological neural networks.

## 1. Introduction

Precise repetitions of neural activity patterns may serve as an infrastructure for numerous neural functions including sensory processing, motor control, and cognition. Synfire chains have been proposed as a fundamental network structure of the nervous system, which can guarantee a fixed level of network activity while allowing to learn and reproduce complicated spatio-temporal firing patterns (Abeles, 1982). Precise neural firing patterns have been found in many brain areas such as the songbird premotor nucleus (Hahnloser et al., 2002) and motor cortex of behaving monkeys (Prut et al., 1998; Shmiel et al., 2006). Studies on isolated neocortical microcircuits have revealed that spontaneous activity, mediated by a combination of intrinsic and circuit mechanisms, can be temporally precise in the absence of sensory stimulation (Mao et al., 2001; Luczak et al., 2007).

There is great interest in understanding how cortical circuits could acquire and maintain synfire-chain-like structures to give rise to relevant computations. Spike timing-dependent plasticity (STDP) has been proposed as a relevant mechanism in previous studies. Hertz and Prugel-Bennett (1996) tried to develop a synfire chain in a random network by introducing a Hebbian learning rule with one-step delay and *n*-winner-take-all dynamics. Successful learning required that the same training stimulus was shown to the system repeatedly. These stimuli, represented as sequences of activation patterns, determined the network dynamics which in turn determined the network connectivity due to STDP and other learning rules. The external stimuli were crucial for the synfire chain formation, because these stimuli generally drove the firing sequence of groups of neurons. Along similar lines, Levy et al. (2001) studied networks in the distributed synchrony activity mode whose dynamics depended on an STDP learning rule and external input. Doursat and Bienenstock (2006) proposed an approach in which a set of seed neurons, a variant of spatiotemporal input, was also found essential for the growth of synfire chains. Similarly, Jun and Jin (2007) investigated an approach that also adopted suprathreshold external input. Hosaka et al. (2008) found that STDP provides a substrate for igniting synfire chains by spatiotemporal input patterns. Clopath et al. (2010) proposed a model of voltage-based STDP with homeostasis behaving similar to a triplet STDP (Pfister and Gerstner, 2006), which could develop variable connectivity patterns. Bourjaily and Miller (2011) studied the incorporation of structural plasticity with a rate-dependent (triplet) form STDP (Pfister and Gerstner, 2006) and the effect on motifs and distribution of synaptic strengths. Kunkel et al. (2011) suggested that biologically motivated plasticity mechanisms in the balanced random network model might lead to the development of feed-forward structures. Other recent approaches employed both different variants of STDP rules and spatiotemporal patterns of stimulation (Iglesias and Villa, 2008; Fiete et al., 2010; Waddington et al., 2012).

Overall, these previous works seem to suggest that the development of synfire chains requires either fine-tuning of model parameters, strong topological constraints on network connectivity, or guidance from strong spatiotemporally patterned training inputs. Here, we show that these limitations can be overcome in a network which combines STDP with additional plasticity mechanisms. We show that synfire chains form spontaneously from randomly initialized self-organizing recurrent networks (SORNs) in the absence of any structured external inputs.

Previous work has shown that SORNs with binary units can learn interesting representations of temporal sequences of sensory inputs (Lazar et al., 2009). Furthermore, we have shown that SORNs reproduce experimental data on the statistics and fluctuations of synaptic connection strengths in cortex and hippocampus, offering a plausible explanation for the experimentally observed approximately log-normal distribution of synaptic efficacies (Zheng et al., 2013). The networks self-organize their structure through a combination of STDP, homeostatic synaptic scaling, structural plasticity, and intrinsic plasticity of neuronal excitability. During network development, the topology adapts as STDP eliminates synaptic connections while structural plasticity adds new ones at a low rate. Meanwhile, the other plasticity mechanisms ensure that the network dynamics remains in a healthy regime.

Here we study the formation of synfire chains in such networks. The networks are initialized with a sparse random connectivity structure and go through dramatic changes in topology with a strong tendency to develop feed-forward motifs. These motifs eventually dominate sub-graph patterns as the network enters into a stable phase where connectivity stays roughly constant. Beyond a simple single feed-forward synfire chain structure, we find multiple ring-shaped chains within one network. The sizes of coactive pools of neurons are influenced by network parameters such as the average firing rate of the excitatory neurons. These results hold true over a wide range of parameters as long as the network operates in a “healthy regime,” supporting the view that synfire chains might be a robust consequence of network self-organization driven by multiple plasticity mechanisms. Overall, our model suggests that the combined action of multiple forms of neuronal plasticity may play an important role in shaping and maintaining cortical circuits and their dynamics, and stereotyped connectivity patterns could arise from the interplay of different plasticity mechanisms at the circuit level.

## 2. Materials and methods

The network model is identical to the one used by Zheng et al. (2013). It is composed of *N^E^* excitatory and *N^I^* = 0.2 × *N*^*E*^ inhibitory threshold neurons connected through weighted synaptic connections. Generally, *W_ij_* is the connection strength from neuron *j* to neuron *i*. *W^EI^* denotes inhibitory to excitatory connections, while *W^EE^* and *W^IE^* denote excitatory-to-excitatory and excitatory-to-inhibitory connections, respectively. The *W^EE^* and *W^EI^* are initialized as sparse random matrices with connection probabilities of 0.1 and 0.2, respectively.

Connections between inhibitory neurons and self-connections of excitatory neurons are not allowed. The *W^IE^* connections are all-to-all and remain fixed at their random initial values which are drawn from a uniform distribution and are then normalized such that the sum of connections entering a neuron is one.

The binary vectors *x(t)* ∈ {0, 1}*^N^E^^* and *y(t)* ∈ {0, 1}*^N^I^^* denote the activity of the excitatory and inhibitory neurons at time step *t*, respectively. The network state at time step *t* + 1 is given by

(1) $$x_{i}(t+1)=\Theta \left(\sum_{j=1}^{N^{E}}W_{ij}^{EE}(t)x_{j}(t)-\sum_{k=1}^{N^{I}}W_{ik}^{EI}(t)y_{k}(t)-T_{i}^{E}(t)+\xi_{E_{i}}(t)\right),$$

(2) $$y_{i}(t+1)=\Theta \left(\sum_{j=1}^{N^{E}}W_{ij}^{IE}x_{j}(t)-T_{i}^{I}+\xi_{I_{i}}(t)\right).$$

The *T^E^* and *T^I^* represent threshold values for the excitatory and inhibitory neurons, respectively. They are initially drawn from a uniform distribution in the interval [0, *T^E^*_max_] and [0, *T^I^*_max_]. Θ(·) is the Heaviside step function. ξ*_E_i__* and ξ*_I_i__* are white Gaussian noise processes with μ_ξ_ = 0 and σ^2^_ξ_ ∈ [0.01, 0.05]. Here one time step corresponds roughly to the duration of an STDP “window.”

The set of *W^EE^* synapses adapts via a simplified causal STDP rule, as reported experimentally (Markram et al., 1997; Bi and Poo, 1998),

(3) $$\Delta W_{ij}^{EE}(t)=\eta_{\text{STDP}}\left(x_{i}(t)x_{j}(t-1)-x_{i}(t-1)x_{j}(t)\right)\;.$$

η_STDP_ is the learning rate. Note that synaptic weights are eliminated if they would become negative due to this rule. To compensate for the loss of synapses, a structural plasticity mechanism adds new synaptic connections between excitatory cells at a small rate. Specifically, with probability *p_c_* = 0.2 a new connection (strength set to 0.001) is added between a randomly chosen pair of unconnected excitatory cells. This models the constant generation of new synaptic contacts observed in cortex and hippocampus (Johansen-Berg, 2007; Yasumatsu et al., 2008).

The incoming excitatory connections to an excitatory neuron are normalized at each time step such that their sum stays constant (Bourne and Harris, 2011). This is achieved by scaling the synapses multiplicatively (Turrigiano et al., 1998; Abbott and Nelson, 2000):

(4) $$W_{ij}^{EE}(t)\leftarrow W_{ij}^{EE}(t)/\sum_{j}W_{ij}^{EE}(t)\;.$$

A homeostatic (intrinsic) plasticity rule maintains a constant average firing rate in every excitatory neuron,

(5) $$T_{i}^{E}(t+1)=T_{i}^{E}(t)+\eta_{\text{IP}}\left(x_{i}(t)-H_{i}^{\text{IP}}\right),$$

where η_IP_ is the adaption rate and the target firing rates *H^IP^^i^* of individual neurons are drawn from a uniform distribution in [μ_IP_ − σ_HIP_,μ_IP_ + σ_HIP_]. In terms of firing rate homeostasis, there are very fast refractory mechanisms which prevent very high firing rates, and there is somewhat slower spike rate adaptation and very slow intrinsic plasticity as seen in some experiments (Desai et al., 1999; Zhang and Linden, 2003). We chose a simple homeostatic regulation of firing rate for our model that can operate relatively fast depending on the choice of the learning rate.

An inhibitory spike-timing dependent plasticity (iSTDP) rule adjusts the weights from inhibitory to excitatory neurons that balances the amount of excitatory and inhibitory drive that the excitatory neurons receive as reported in recent studies (Haas et al., 2006; Vogels et al., 2011, 2013),

(6) $$\Delta W_{ij}^{EI}(t)=-\eta_{\text{inhib}}y_{j}(t-1)\;\left(1-x_{i}(t)(1+1/\mu_{iSTDP})\right),$$

where η_inhib_ is the adaption rate, and μ_i*STDP*_ is set to 0.1 for all the simulations.

Unless otherwise specified, the simulations are conducted using the following parameters. η_IP_ = 0.01, *T^E^*_max_ = 1, *T^I^*_max_ = 0.5, μ_IP_ = 0.1, σ_HIP_ = 0, η_inhib_ = 0.001, σ^2^_ξ_ = 0.01. Parameter η_STDP_ decreases monotonically as network size *N^E^* increases, and η_STDP_ = 0.004, 0.002 and 0.001 for *N^E^* ∈ [200,400], [600,800] and [1000,1200] respectively.

## 3. Results

### 3.1. Feed-forward motifs dominate subgraph patterns

**Figure 1:**
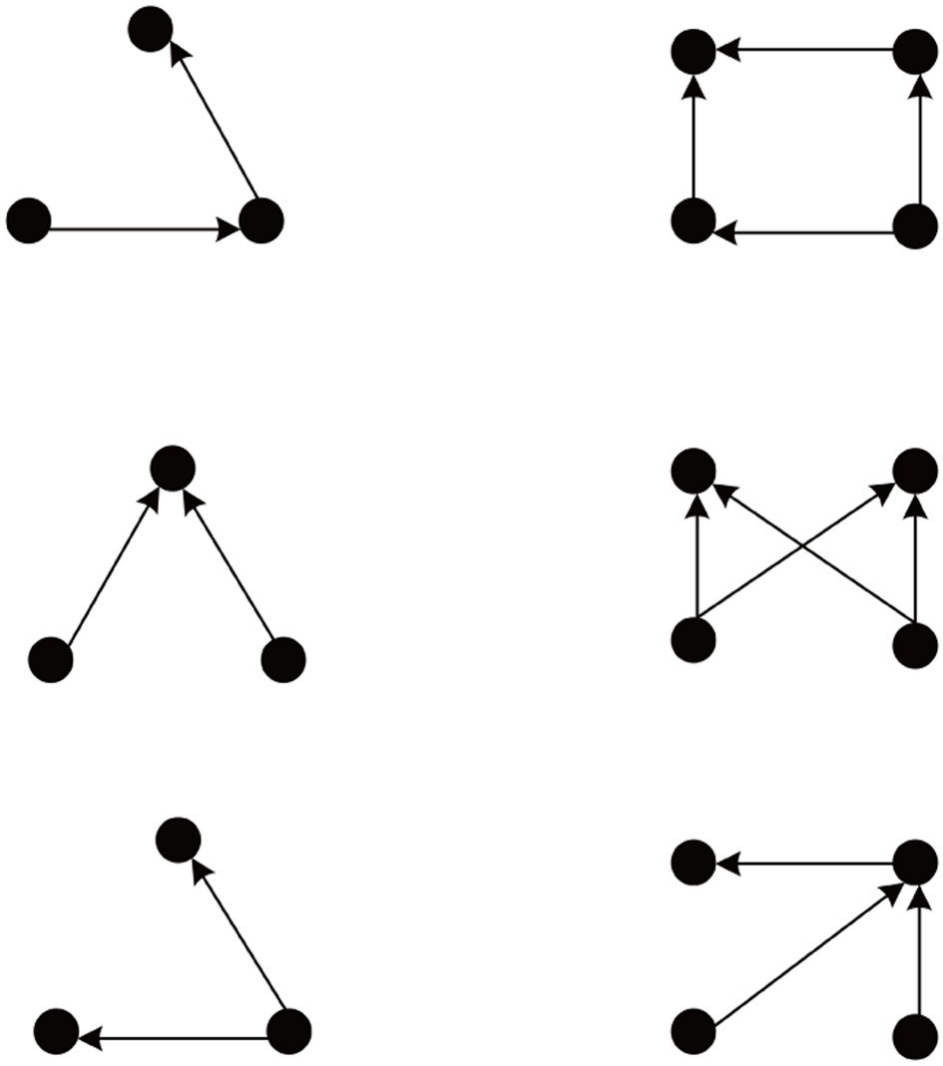
**Abundant network motifs observed in the stable regime. Left column:** Three neuron motifs. **Right column:** Four neuron motifs. All detected motifs have a feed-forward structure compatible with a network topology dominated by synfire-chains.

We simulate 10 networks, and initial weights of each network are randomly selected from uniform, Gaussian, delta (all weights identical), or exponential distributions. After weight initialization, each such network is examined on 10 different sets of network evolution parameters, such as neuron number, learning rates, neuron firing rates, etc. The network connectivity changes due to the action of the different plasticity mechanisms. As observed in Zheng et al. (2013), the network goes through different phases characterized by the number of excitatory-to-excitatory connections present in the network. Eventually, it enters a stable regime where connectivity stays roughly constant. For such stabilized networks we use the Fanmod software (Wernicke, 2005) and its computation of a *p*-value to analyze network motifs involving 3 and 4 neurons. Here the *p*-value of a motif is defined as the number of random networks in which it occurred more often than in the original network, divided by the total number of random networks. Therefore, *p*-values range from 0 to 1, and the smaller the *p*-value, the more significant is the abundance of the motif. The frequency of a motif occurring in 100 simulated SORN networks is compared to the mean frequency of the motif occurring in 1000 random networks with identical connection probability. We found the network motifs are organized into two distinct groups with *p*-value = 0 and *p*-value = 1. Figure 1 shows the group of motifs always with *p*-value = 0, all of which reveal a feed-forward structure consistent with a synfire-chain topology.

### 3.2. Evolution of network connectivity

**Figure 2:**
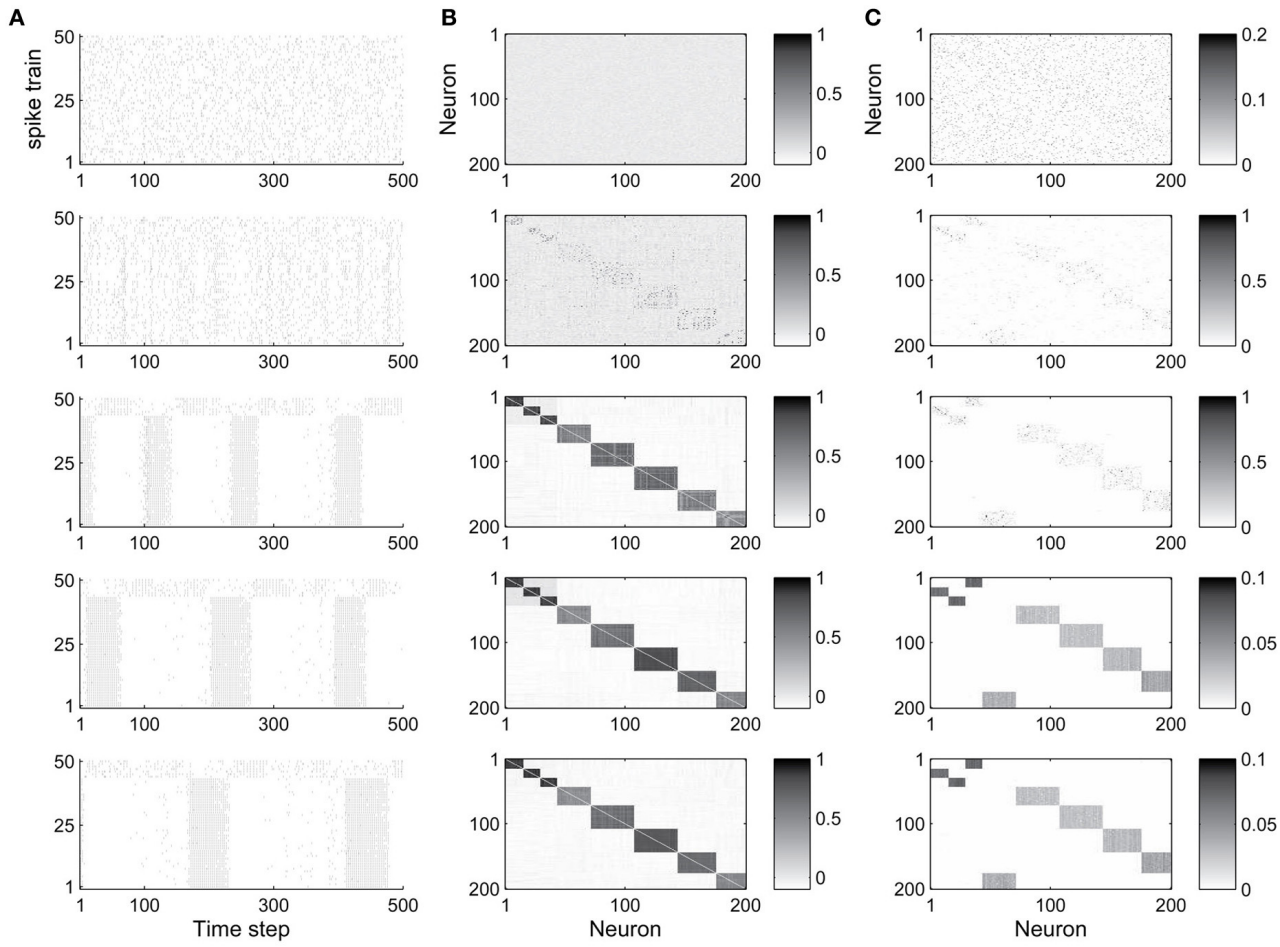
**Evolution of network dynamics and connectivity. (A)** Spike trains of a set of 50 neurons during different phases of network development. **(B)** Activity correlations between all excitatory units. **(C)** Excitatory connectivity. Gray value represents excitatory synaptic strength as illustrated in the scale bar. From top to bottom, the five rows show data starting from the 1st (initial phase), 20,000th (early phase), 500,000th (intermediate phase), 1000,000th (late phase), and 4000,000th (final phase) time step, respectively.

The abundance of feed-forward network motifs among groups of 3 and 4 neurons during the stable phase of network evolution already suggests that the network may be forming synfire-chain like structures. To investigate this, we studied the evolution of the network’s activity patterns and connectivity during its self-organization. Figure 2 shows an example. In Figure 2A we plot the activity of the first 50 neurons during short 500 time step intervals taken at five different time points of the network’s evolution. Excitatory neurons are sorted in all recorded networks according to their activity correlations in the last recorded network (in the stable phase). Thus neurons that are highly correlated during the stable phase are plotted in neighboring rows. While the network initially exhibits quite irregular activity, it spontaneously forms highly structured activity patterns as it develops (also see Figure S1 in the supplementary material, which shows example cross-correlograms of different pairs of neurons). In the particular case shown here, the network forms two subsets of neurons which alternate in exhibiting phases of high firing rates.

Figure 2B shows the evolution of firing correlations among all excitatory neurons in the network. The network forms 8 distinct pools of neurons, with neurons of each pool exhibiting highly synchronized firing. The excitatory weight matrix shown in Figure 2C reveals that the network develops two independent circular synfire chains, which we will refer to as synfire-rings. The layers of synfire rings are identified automatically by applying a threshold to the neurons’ activity correlations. Due to noise and the interaction of multiple forms of plasticity, a neuron’s activity maintains a certain degree of randomness, which leads to positive but non-uniform correlations in each layer. As a result there are some neurons with relatively weaker correlation in each layer in most cases.

In the given example, the first synfire-ring comprises 3 smaller pools of neurons (total of 43 neurons), the second synfire ring comprises 5 larger pools of neurons (total of 157 neurons). The two synfire-rings correspond to two transiently stable activity patterns. As shown in Figure 2, activities of the first 43 and remaining 7 neurons, which belong to different rings, are roughly complementary. If one synfire ring becomes active, it tends to activate the inhibitory neurons and thereby suppress activity in the other synfire ring. After a while, however, the intrinsic plasticity mechanism will increase the firing thresholds of neurons belonging to the active synfire-ring and decrease the firing thresholds of the inactive synfire-ring. Over time, this destabilizes the active synfire-ring and eventually leads to the suppressed synfire-ring taking over. The strong competition between the synfire rings is due to the widespread inhibition with each inhibitory unit receiving input from all excitatory cells in the network and projecting randomly to one fifth of the excitatory cells (compare Methods).

### 3.3. Influence of target firing rate on sizes of neuronal pools

We next investigate how the sizes of neuronal pools and their connectivity depend on the target firing rates of the neurons in a 200 excitatory neuron network with fixed initial connectivity. The parameter *H*^IP^*_i_* sets the target firing rate for the *i*-th excitatory neuron. These target firing rates are drawn from a uniform distribution in [μ_IP_ − σ_HIP_, μ_IP_ + σ_HIP_].

**Figure 3:**
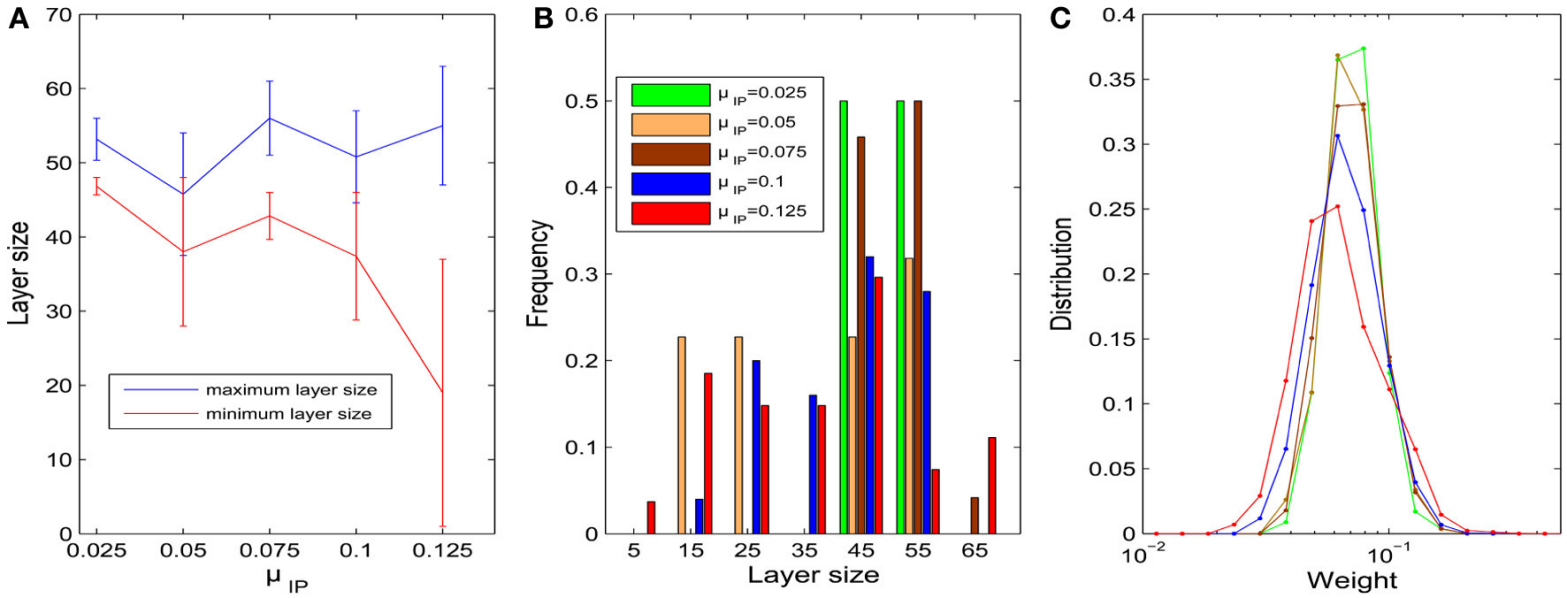
**Influences of parameter μ_IP_ on the layer/pool size (σ_HIP_ = 0). (A)** Changes of maximum and minimum layer size as μ_IP_ varies from 0.025 to 0.125. Error bars represent SD. **(B)** Histograms of layer sizes. **(C)** Distributions of synaptic weight strengths in the stable phase are all lognormal-like. Note that x-axis is log-scale and color index is identical with **(B)**.

We first fix σ_HIP_ = 0 and study the influence of the target firing rate μ_IP_. As the target firing rate of the neurons increases, the variability of the sizes of neuronal pools increases. Figure 3A plots the average maximum and minimum pool sizes as a function of μ_IP_. For large μ_IP_, the maximum layer size tends to get bigger and the minimum layer size tends to be smaller. In addition, the variability of the maximum and especially the minimum layer sizes tends to be largest for the biggest μ_IP_. Figure 3B compares the histograms of pool sizes for different μ_IP_. The distribution is very narrow for small μ_IP_ (green bars corresponding to μ_IP_ = 0.025) and very broad for large μ_IP_ (red bars corresponding to μ_IP_ = 0.125). In all cases, the final distribution of synaptic strength is lognormal-like which means some weights are way stronger than others. This is shown in Figure 3C, which plots this distribution for different μ_IP_.

**Figure 4:**
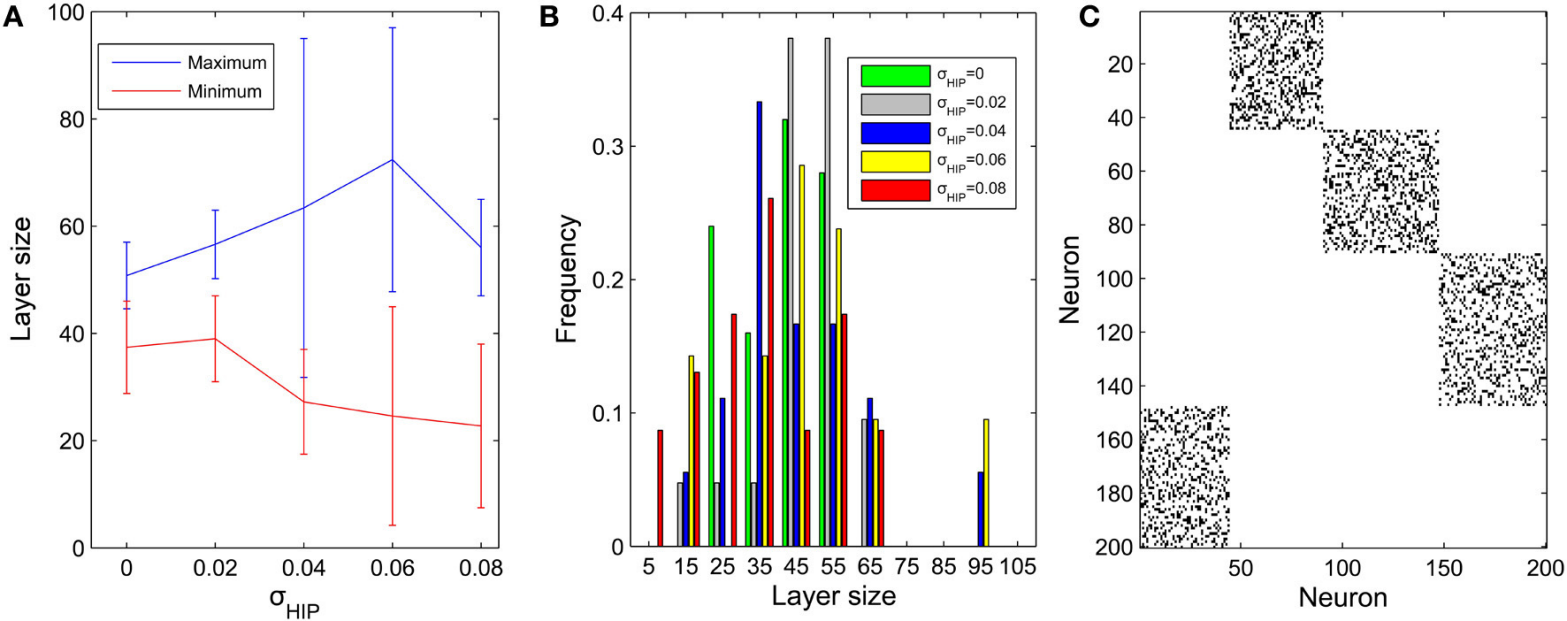
**Influences of σ_HIP_ on the layer size (μ_IP_ = 0.1). (A)** Changes of maximum and minimum layer size as σ_HIP_ varies from 0 to 0.08. Error bar is SD. **(B)** Distributions of pool size. **(C)** Typical example of network connectivity in the stable phase for σ_HIP_ = 0.06. Black dots represent synapses whose weights are bigger than 0.01. The network has developed into a single synfire ring with 4 pools of neurons of sizes 44, 57, 53, 46.

We next fix μ_IP_ = 0.1 and study the effect of the interval size σ_HIP_ of the target firing rates. In a similar way, the diversity of pool sizes grows as σ_HIP_ increases. This holds true for σ_HIP_ ≤ 0.06 as shown in Figure 4A. However, as σ_HIP_ increases more and more neurons are close to silent. The minimum target firing rate of some excitatory neurons is as small as ~0.02 when σ_HIP_ reaches 0.08. These neurons barely fire during the network evolution and barely contribute to structuring the network. Therefore, the effective network size is reduced as σ_HIP_ is increased. This may explain why the variability in pool sizes shrinks when σ_HIP_ grows to 0.08. Figure 4B compares the distribution of pool sizes for different values of σ_HIP_. The greatest spread of the distribution is obtained for an intermediate value of σ_HIP_ = 0.06. Figure 4C shows an example of an excitatory weight matrix in the stable regime for σ_HIP_ = 0.06. The network has developed a single synfire ring with 4 pools of neurons whose sizes range from 44 to 57.

### 3.4. Influence of network size on synfire ring structure

**Figure 5:**
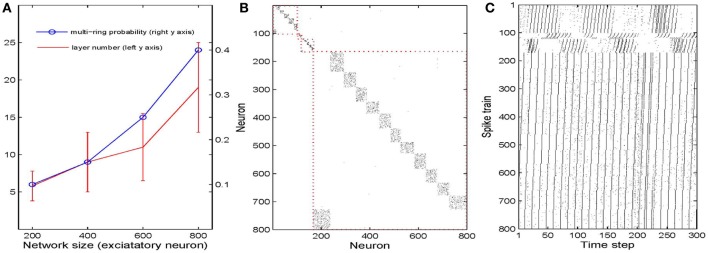
**Influences of network size. (A)** Changes of multi-ring probability and layer number as network size varies from 200 to 800. Error bars are SD. **(B)** Typical example of network connectivity with four synfire rings in the stable phase of a 800 excitatory neuron network. Black dots represent synapses whose weights are bigger than 0.01. **(C)** Spike trains of the neurons in **(B)**.

We next study how the number of synfire rings and the number of neuronal pools or layers depends on the overall network size. To this end, we simulate 40 networks with 200–800 excitatory neurons. As a first measure of network structure we define the number of layers present in the network. Figure 5A plots this number as a function of network size (red curve). Not surprisingly, the number of neuronal pools increases as the network gets bigger. As a second index of network structure we measure the fraction of networks of a given size that develop multiple synfire rings. As shown in Figure 5A (blue curve) this fraction increases with network size. For networks of 800 neurons it already reaches a value of 0.4 and the increase with network size seems to be faster than linear for the range of sizes considered. Figure 5B shows a typical example of the excitatory weight matrix in a network with 800 neurons and μ_IP_ = 0.1, σ_HIP_ = 0. This network has developed 4 synfire rings of different sizes. Note that the second one from the top is very small. In Figure 5C it is easier to identify it. The sizes of the pools are fairly consistent within a single synfire ring (mean *SD* is 4.5) but can vary widely across synfire rings (*SD* is 26.6). The biggest ring in Figure 5B has 12 pools, so if activity runs around in this circle, each neuron is activated only every 12th time step, which is less than intrinsic plasticity wants (compare Methods). As shown in Figure 5C, the biggest ring is roughly active all the time, and unlike the synfire rings in Figure 2, the network could start multiple rings simultaneously. It is worth noting that we achieve the synfire ring structure under a wide range of parameters, excitatory neuron number being one of them. We also run a few simulations with 1000 and 1200 excitatory neurons, which also develop synfire rings (see Figures S2, S3 in supplementary material).

### 3.5. Mechanisms of synfire ring formation

With all forms of plasticity present, the network will develop synfire rings spontaneously and robustly over a large range of parameters as long as the network operates in a healthy regime. The results are fully in line with our previous work since we use same network as Zheng et al. (2013), where we discuss in detail the necessity of the different plasticity mechanisms for the networks behavior. So how do these (circular) feed-forward structures come about?

**Figure 6:**
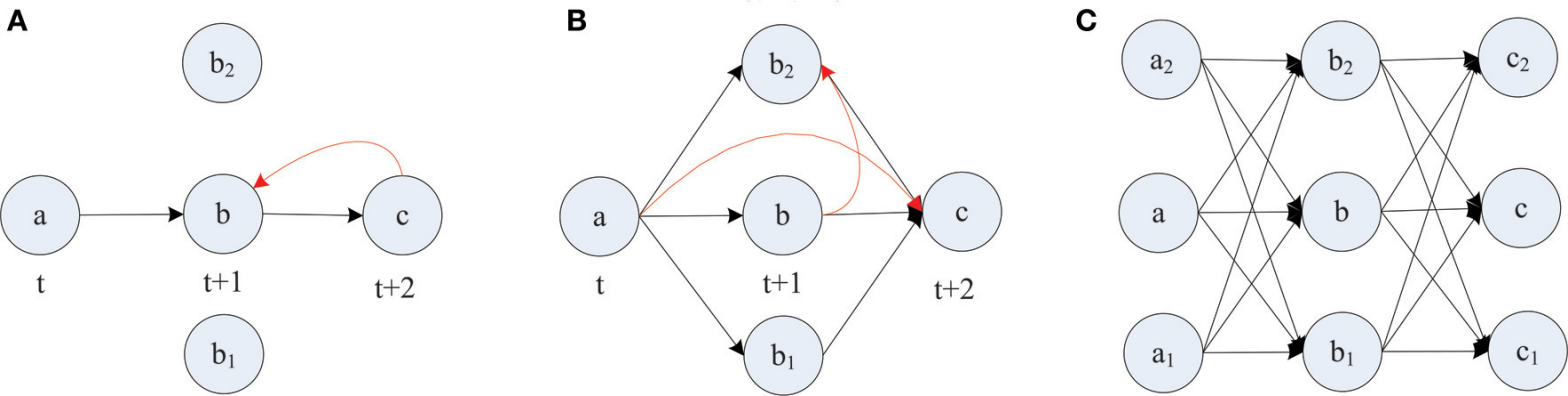
**Layered synfire chain structure formation**. **(A)** Removal of reciprocal connection. **(B)** Establishment of parallel pathways. **(C)** Formed synfire chain. Red arrows represent spurious synapses that are inconsistent with the developing synfire chain structure. Black arrows represent synapses that conform to the synfire route.

**Figure 7:**
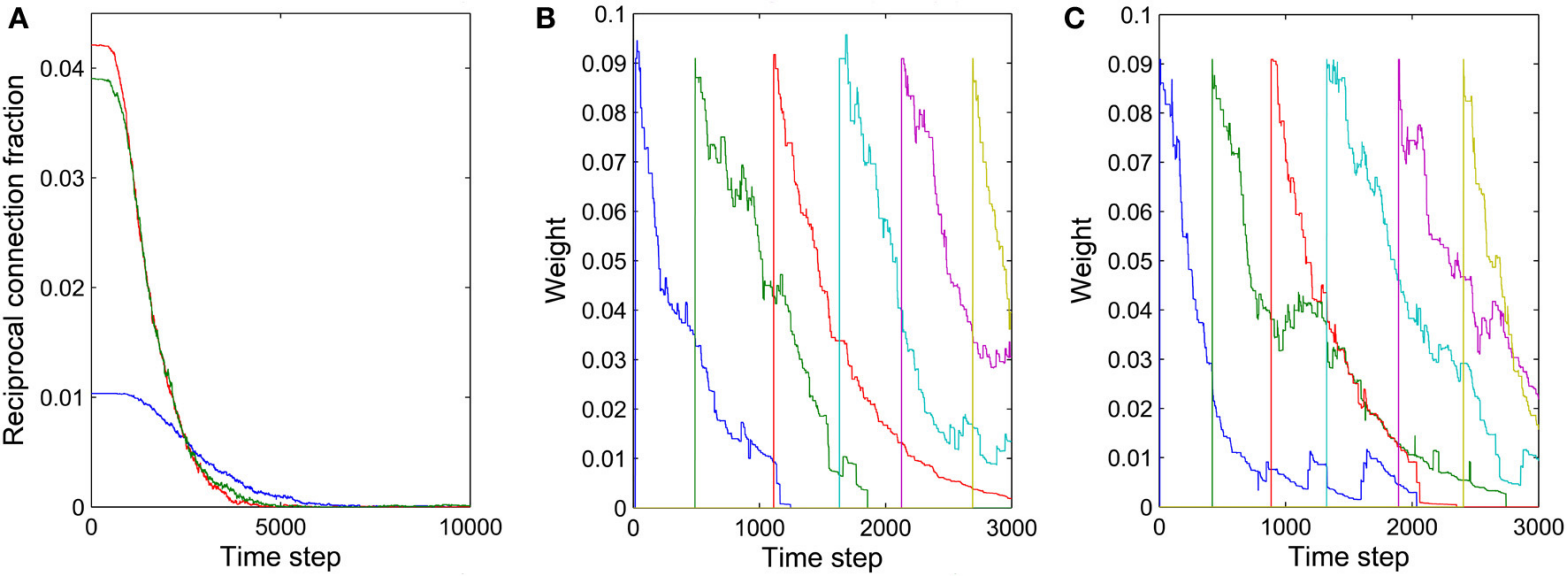
**Illustration of two mechanisms contributing to synfire ring formation. (A)** Fraction of reciprocal connections as function of time for three independent networks with (*N^E^*)^2^ as the denominator. **(B)** Synaptic weights of connections within one layer as a function of time. **(C)** Synaptic weights of connections from one layer to the second next layer as a function of time. The connections in **(B,C)** were manually added approximately every 500 time steps.

The formation of synfire-chains can be understood as a process of network self-organization driven largely by the STDP rule. Figure 6 illustrates the process. Consider as an example a strong feed-forward chain from a unit *a* to a unit *b* and on to a unit *c*. According to this structure, there is a high probability that *a*, *b*, and *c* fire in three successive time steps. Standard STDP rules, including the one we are using here, will strengthen the connections in the feed-forward direction and weaken the reverse connections such as the red synapse in Figure 6A. This is because of the nature of the STDP rule, which potentiates “causal” firing patterns (pre before post) and depresses “acausal” firing patterns (post before pre). As shown in Figure 7A, the fraction of bidirectional connections plummets during the first stage of network evolution. Thus, a first relevant mechanism in synfire ring formation is the *removal of reciprocal connections*.

A second mechanism in synfire ring formation is the *establishment of parallel pathways*. Consider two units *b*_1_ and *b*_2_ which also happen to be strongly innervated by *a* (see Figure 6B). Because of this, they will tend to be synchronously active with unit *b* and their activity will be reliably followed by activation of unit *c*. Because of this correlation structure (*b*_1_ and *b*_2_ likely being active in the time step before *c*) the weights from *b*_1_ and *b*_2_ onto *c*, if present, will have a strong tendency to get potentiated. Thus, STDP will potentiate the “missing” connections from *b*_1_ and *b*_2_ onto *c* establishing additional parallel pathways connecting *a* and *c*. In order for STDP to be able to strengthen these connections, they have to either be present from the beginning or become added by the structural plasticity. With this mechanism operating not just at the level of *b* but at all levels of the network, synfire-chains will develop (see Figure 6C). Due to the homeostatic activity regulation, at each time step a certain fraction of neurons in the network will tend to be active. This implicitly regulates the range of layer sizes and limits the breadth of the growing chain. Due to the synaptic scaling, every neuron in a layer receives a certain amount of synaptic input. Moreover, since the network has only a finite number of units and each unit tries to maintain a certain average activity level such that activity cannot die out, it is inevitable that such a chain eventually terminates or connects back to itself thereby forming a synfire ring. A ring-like structure has a competitive advantage against a terminating chain during the formative stage of network development, because a synfire ring will reactivate itself while a terminating chain cannot.

STDP alone can not depress existing synapses that are incompatible with the emerging synfire ring structure. For example, connections within one layer of neurons or connections jumping ahead beyond the immediate next layer (compare red synapses in Figure 6B) remain unaltered under perfect synfire chain activity. However, the synaptic scaling mechanism gradually depresses these connections to very small values as the other weights on the synfire route are potentiated. Thus, another relevant mechanism in synfire ring formation is the *competition among synaptic weights onto the same target neuron*.

The synaptic scaling mechanism we use does not remove any such “spurious” synapses, however. This is achieved by STDP. Due to intrinsic membrane noise of the neurons and fluctuations of intrinsic excitability and inhibitory drive, the network’s activity always maintains a random component—even in its stable phase (compare Figure 2A). As a consequence, neuron *a* in Figure 6 could fire right after *c*, which would lead to the removal of a sufficiently depressed spurious connection from *a* to *c*. Such events occur only rarely but they suffice to eliminate such spurious connections if they have already been depressed by synaptic scaling. Thus, a final mechanism in synfire ring formation is the *removal of spurious connections due to random activity fluctuations*. To illustrate this effect, we manually added new connections with rather strong weights of value 0.1 within one layer and between one layer and the layer two steps ahead. These manually added new connections are even stronger than ~70% of the existing connections. Figures 7B,C show the fate of these manually inserted connections that are inconsistent with the dominant synfire-ring structure: within a few thousand time steps their weights decrease to zero as a result of competition among synapses and STDP driven by random activity fluctuations.

**Figure 8:**
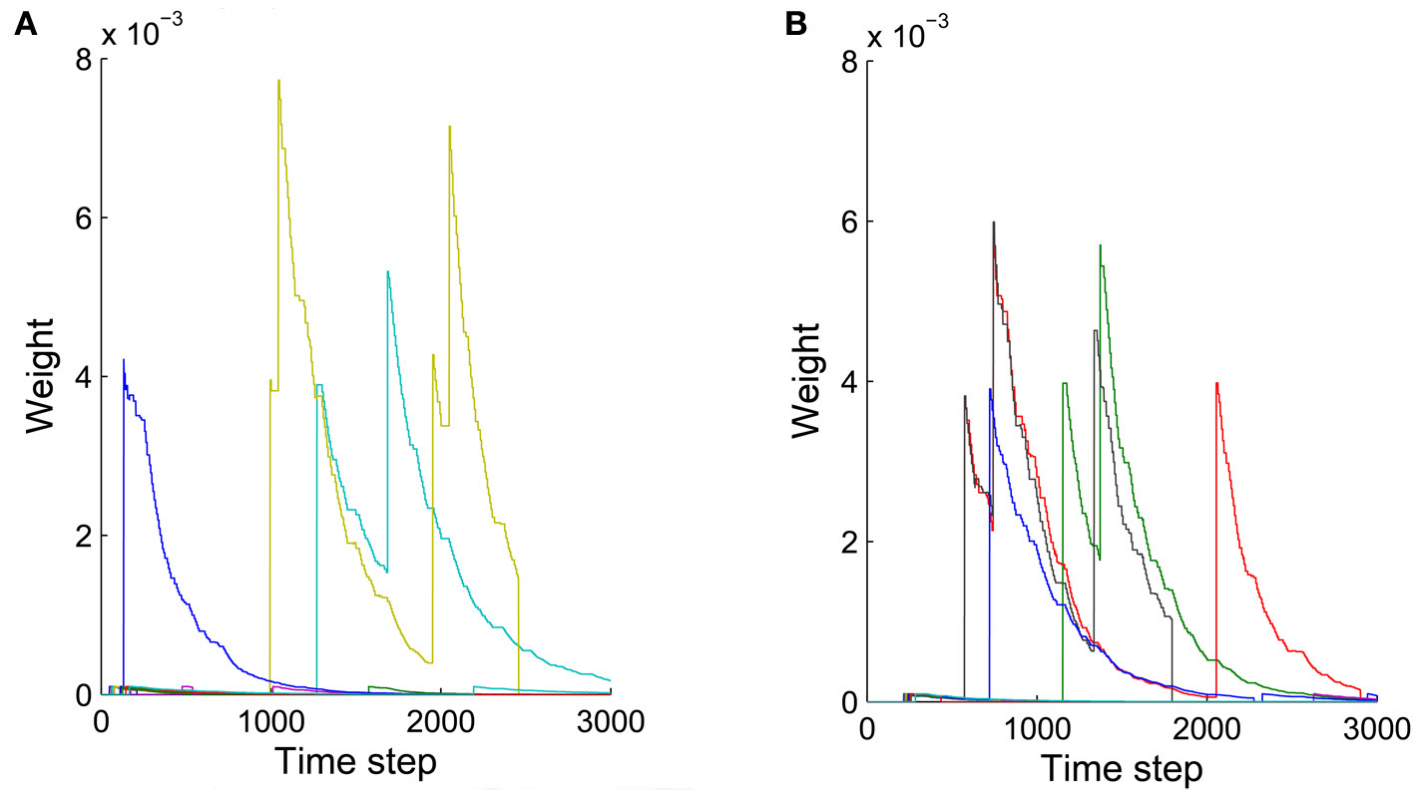
**Weights of synaptic connections that have been added by structural plasticity during the stable phase as a function of time**. Colors represent different synapses. **(A)** Ten synapses added on the synfire route. **(B)** Ten synapses that are not on the synfire route. Note that in both cases some of the synapses are eliminated immediately after birth.

The precise outcome of the overall network self-organization depends on the initial conditions (initial network structure and connection weights) and the random activity fluctuations. Feed-forward connections between synfire layers go through strong competition during network evolution as a result of synaptic scaling. Connections that start out strong or are added early have an advantage in this competition and are less prone to removal due to random activity fluctuations. Synapses added in later phases of the network’s evolution are more fragile, which contributes to the stability of already formed synfire rings. Figure 8 shows examples of weight changes of new synapses that have been added through structural plasticity during the network’s stable phase. In Figure 8A we plot synaptic connections that are off any existing synfire ring structure. These connections are removed comparatively quickly. Figure 8B illustrates the fate of newly added synaptic connections that are congruent with an existing synfire ring, i.e., they connect a neuron from one pool to a neuron in the successor pool. Interestingly, even these connections tend to be removed eventually. Due to synaptic scaling, they have to compete with many other connections along the synfire ring, which limits their growth and makes them prone to elimination due to random activity fluctuations. It should be clarified that Figures 7B,C (unlike Figure 7A) and Figure 8 all study the network in its stable phase. That is the synfire chain is already formed, which is analogous to a prewired synfire chain. In all of these cases, the synfire chain is indeed restored after our perturbation. Figures 7B,C, 8B are similar, but they are different in terms of new synapse weight and position definition.

It should be mentioned that the network becomes rather rigid only after synfire chains have been formed. This is important to maintain the stability of synfire chains. At the beginning of network evolution, however, newer synapses are freely competing. During the development from a randomly initialized network to synfire chains, many new synapses are added and stabilized. Besides that, newer synapses could also survive in synfire chains when the network is driven by appropriate strong structured external input (not shown).

As mentioned above, every plasticity mechanism is important for the development of synfire structure. In simulations, we did not observe the formation of synfire rings in networks without synaptic normalization, structural plasticity or STDP of the excitatory connections. Intrinsic plasticity and inhibitory STDP both try to maintain a low average firing rate of excitatory cells, and the formation of synfire structure relies on the presence of both of them. If we switch off one of them, the network suffers from big activity fluctuations from time to time, which usually stop the formation of synfire structure or lead to abnormal network structures exhibiting both extra-large and single-neuron layers (Figure S4 in the supplementary material).

## 4. Discussion

Since their introduction by Abeles (1982), synfire-chains have been the subject of intense experimental and theoretical investigation. Here we have studied the spontaneous formation of synfire-chains in self-organizing recurrent neural networks (SORNs) shaped by multiple plasticity mechanisms. These networks have been shown to learn effective representations of time-varying inputs (Lazar et al., 2009) and to reproduce data on the statistics and fluctuations of synaptic connections strength in cortex and hippocampus (Zheng et al., 2013). There is also some empirical evidence for their ability to approximate Bayesian inference (Lazar et al., 2011). Despite their simplicity in terms of using binary threshold units operating in discrete time, they have been a useful tool for studying the interaction of different forms of plasticity at the network level. In the present study, we have combined simple spike-timing-dependent plasticity (STDP) rules for excitatory-to-excitatory and inhibitory-to-excitatory connections with a synaptic normalization and firing rate homeostasis of excitatory units. Furthermore, a structural plasticity rule created new excitatory-to-excitatory connections at a low rate.

The initial connection probability of excitatory to excitatory connections is set to 0.1, which falls in the biologically plausible range. In simulations, we couldn’t decrease this probability further, otherwise the network decomposed into unconnected smaller ones. In some cases, the structural plasticity may re-connect these pieces, but not all the time. Generally, it is hard to draw any conclusion from the simulations of such unhealthily initialized networks. In the other extreme, we can increase initial connection probability all the way to a fully connected network and the network will still develop synfire rings.

We found that the STDP mechanism prunes bidirectional connections between pairs of excitatory neurons, which is consistent with previous modeling work (Abbott and Nelson, 2000). It is interesting to note that there maybe layer-specific differences in cortex in terms of the abundance of such bidirectional synaptic connections with layer 5 showing many bidirectional connections in one study (Song et al., 2005), but layer 4-2/3 showing very few (Feldmeyer et al., 2002; Lefort et al., 2009). The pruning of bidirectional connections goes hand in hand with the emergence of feed-forward chains among pools of neurons. This formation of synfire-chains represents a phenomenon of network-self-organization. Partial feed-forward structures between pools of neurons have a tendency to become amplified due to STDP while the homeostatic plasticity mechanisms induce competition among the developing feed-forward structures. These feed-forward chains assume a ring-shaped topology, which we refer to as synfire-rings. We observed that the number of synfire rings, their lengths, and the sizes of their pools are influenced by the distribution of firing rates. The development of “orderly” synfire dynamics in these networks is consistent with previous results indicating a reduction of chaotic behavior in these networks (Eser et al., 2014).

Previous modeling studies on the formation of synfire-chains have used more realistic model neurons and synapses, but have omitted some of the plasticity mechanisms incorporated into the present model. As was shown previously (Zheng et al., 2013), these mechanisms may be essential for explaining critical aspects of cortical wiring such as the log-normal distribution of excitatory-to-excitatory synaptic efficacies and the pattern of fluctuations of synaptic efficacies. It is clear that a long-tailed, highly skewed distribution of synaptic efficacies may strongly affect synfire dynamics, since the simultaneous activation of only few extremely strong synapses may suffice to elicit an action potential in the postsynaptic neuron. In the present study, lognormal-like statistics of excitatory synaptic connections develop robustly in the network (see Figure 3C). To our knowledge, no previous study has investigated synfire dynamics with lognormally distributed excitatory-to-excitatory efficacies. Our model does not only demonstrate synfire dynamics with a biologically realistic distribution of excitatory-to-excitatory synaptic efficacies, it also shows how this distribution and synfire dynamics emerge from fundamental plasticity mechanisms in the absence of any structured input to the network.

Overall, we conclude that the combination of a number of generic plasticity mechanisms is sufficient for the robust formation of synfire chains with synaptic connection statistics matching biological data. Many aspects of our model could be made more realistic. For instance, it will be important to go beyond networks of binary threshold units operating in discrete time steps. We would like to test if similar results can be obtained in more realistic networks of spiking neurons operating in continuous time. Another limitation is that we have assumed identical one time step conduction delays of all synaptic connections. Izhikevich (2006) however found that conduction delays were important for time-locked but not synchronous spiking activity, and managed to generate many more synfire “braids” than the number of neurons in the network. The consideration of heterogeneous conduction delays in a more realistic version of our model is an interesting topic for future work.

## Author contributions

Conceived and designed the experiments: Pengsheng Zheng, Jochen Triesch. Performed the experiments: Pengsheng Zheng. Analyzed the data and plotted the results: Pengsheng Zheng. Wrote the paper: Jochen Triesch, Pengsheng Zheng.

### Conflict of interest statement

The authors declare that the research was conducted in the absence of any commercial or financial relationships that could be construed as a potential conflict of interest.
