# Supplementary material for: Robust development of synfire chains from multiple plasticity mechanisms
Source: Front Comput Neurosci. 2014 Jun 30;8:66. doi: 10.3389/fncom.2014.00066 (PMC4074894; doi:10.3389/fncom.2014.00066)
Supplement: Supplementary file 1 [file Presentation1.PDF]

Supplementary Material

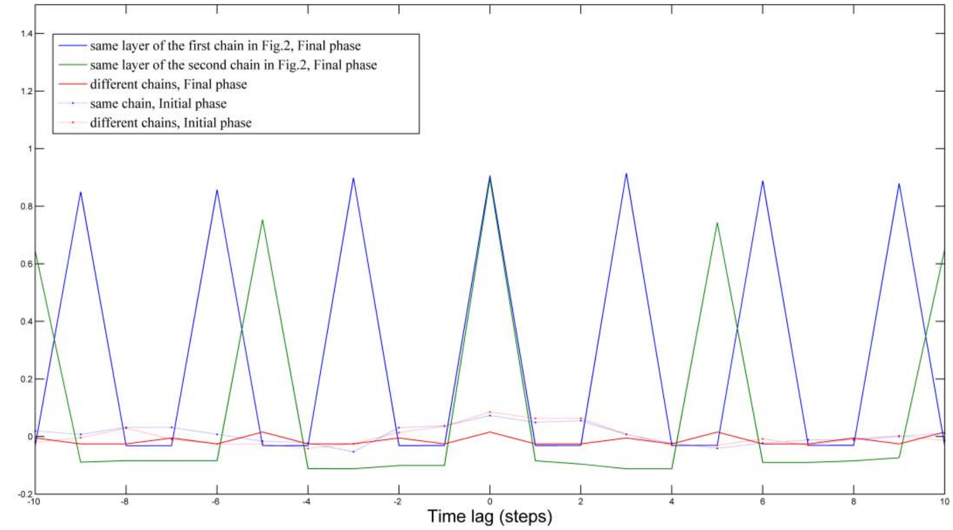

Fig.S1: Cross-correlograms between two typical neurons at the beginning (Initial phase) and end (Final phase) of network evolution

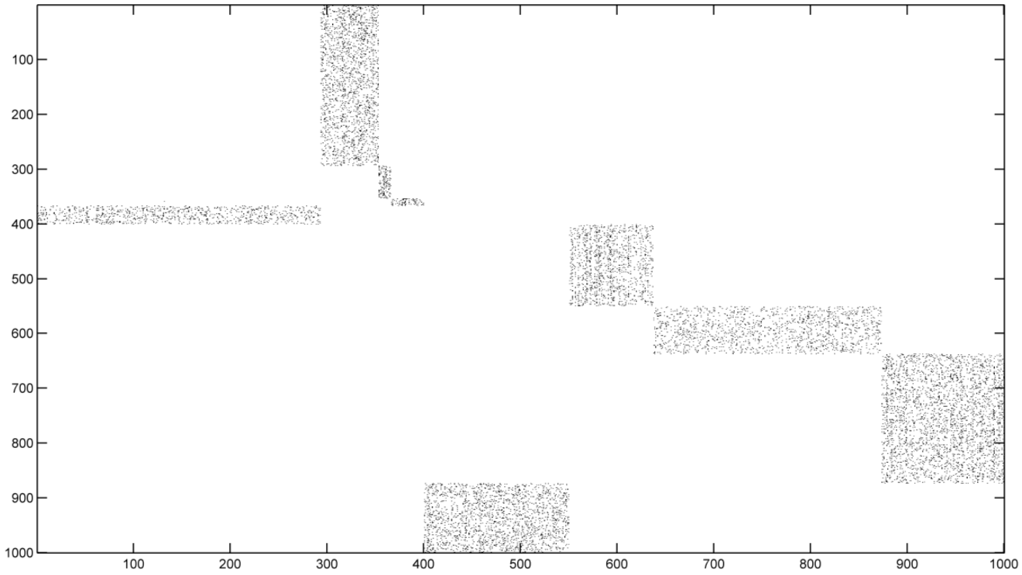

Fig.S2: Example of synfire ring developed in a 1000 excitatory neuron network

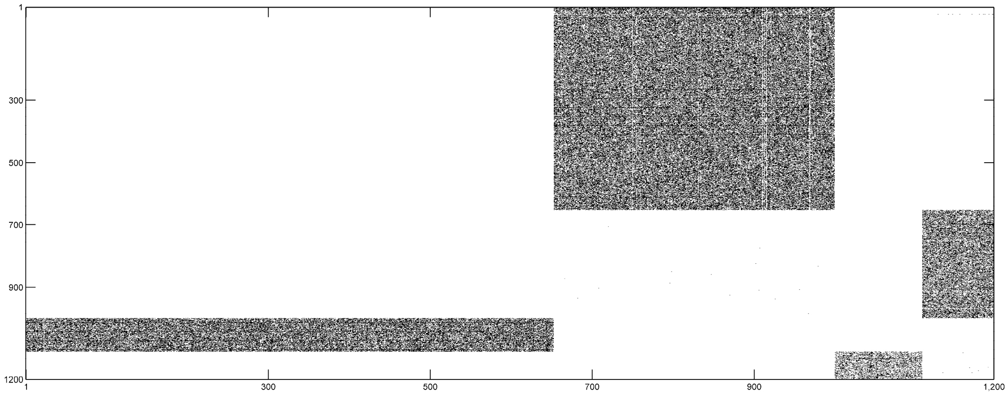

Fig.S3: Example of synfire ring developed in a 1200 excitatory neuron network

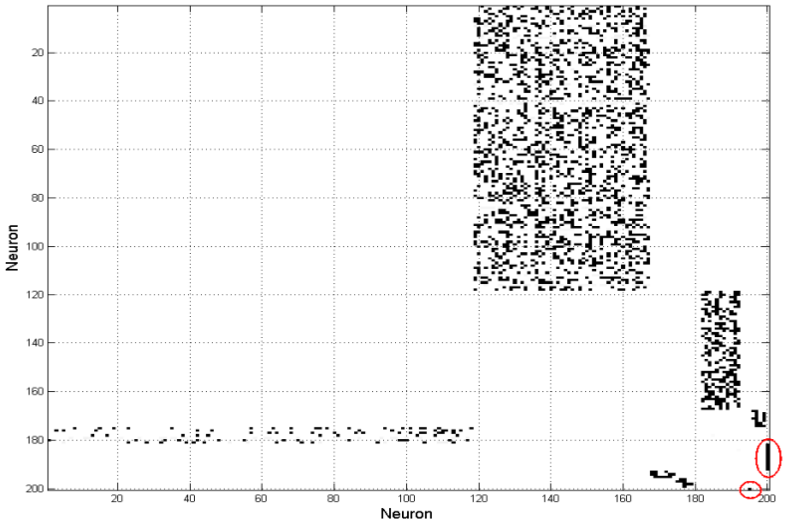

Fig.S4: Network structure after turning off the iSTDP. The network exhibits both extra-large and single-neuron layers. Note: red ellipses mark the single-neuron layers
